# Prior mucosal exposure to heterologous cells alters the pathogenesis of cell-associated mucosal feline immunodeficiency virus challenge

**DOI:** 10.1186/1742-4690-7-49

**Published:** 2010-05-28

**Authors:** Surender B Kumar, Sarah Leavell, Kyle Porter, Barnabe D Assogba, Mary J Burkhard

**Affiliations:** 1 Department of Veterinary Biosciences, The Ohio State University, Columbus, Ohio, USA; 2 Center for Retrovirus Research, The Ohio State University, Columbus, Ohio, USA; 3 Center for Microbial Interface Biology, The Ohio State University, Columbus, Ohio, USA; 4 Center for Biostatistics, The Ohio State University, Columbus, Ohio, USA

## Abstract

**Background:**

Several lines of research suggest that exposure to cellular material can alter the susceptibility to infection by HIV-1. Because sexual contact often includes exposure to cellular material, we hypothesized that repeated mucosal exposure to heterologous cells would induce an immune response that would alter the susceptibility to mucosal infection. Using the feline immunodeficiency virus (FIV) model of HIV-1 mucosal transmission, the cervicovaginal mucosa was exposed once weekly for 12 weeks to 5,000 heterologous cells or media (control) and then cats were vaginally challenged with cell-associated or cell-free FIV.

**Results:**

Exposure to heterologous cells decreased the percentage of lymphocytes in the mucosal and systemic lymph nodes (LN) expressing L-selectin as well as the percentage of CD4+ CD25+ T cells. These shifts were associated with enhanced ex-vivo proliferative responses to heterologous cells. Following mucosal challenge with cell-associated, but not cell-free, FIV, proviral burden was reduced by 64% in cats previously exposed to heterologous cells as compared to media exposed controls.

**Conclusions:**

The pathogenesis and/or the threshold for mucosal infection by infected cells (but not cell-free virus) can be modulated by mucosal exposure to uninfected heterologous cells.

## Background

In the early 1990s, immunization against major histocompatibility complex (MHC) alloantigens was proposed as a potential human immunodeficiency virus (HIV)-1 vaccine strategy [1]. Recently, interest in the potential of alloprotection against HIV-1 transmission has gained new momentum with the findings that allogeneic mismatch may be associated with reduced sexual and vertical transmission.

Animal model vaccine studies suggest that exposure to heterologous antigens play a role in protection against lentiviral infection. In simian immunodeficiency virus (SIV) and feline immunodeficiency virus (FIV) studies, the efficacy of cell-based vaccines has been shown to be, at least in part, due to immune responses against the heterologous cells [2-8]. In the SIV system, this protective mechanism has further been delineated as both humoral and cell-mediated responses against MHC molecules [2-5,9,10]. Consistent with animal model studies are epidemiological reports that support a role for alloantigen driven immune responses in HIV-1 resistance. For example, women who have less common human leukocyte antigen (HLA) types for their region are over-represented in cohorts of sex workers who remain seronegative despite repeated high-risk exposure [11].

While more studies are required to ascertain if susceptibility to HIV-1 infection, disease progression, or both are associated with certain HLA clusters, the protective role for induced immunoreactivity against HLA antigens appears to be well established [1,12,13]. Alloimmune responses can provide both neutralizing antibody [14] and cell-mediated [14-16] antiviral activity against HIV-1 and alloimmunization of women elicits a dose dependent decrease in the susceptibility of CD4+ T-cells to in vitro HIV-1 infection [17]. Similar anti-HLA immune responses have been identified in exposed seronegative sex workers [17-19] and infants born to HIV-1-infected mothers [20].

Alloantigen exposure can directly modulate the production of soluble factors and cell surface receptors. Alloimmunization has been shown to elicit CD8+ T-cell anti-HIV-1 activity as well as production of RANTES, MIP-1α and β [16]. Importantly, sexual contact may be sufficient to induce alloimunization that alters the expression of HIV-1 receptors. This was demonstrated by finding that CD4+ T cells from women with unprotected sexual activity were highly resistant to binding by either CCR5 or CXCR4 strains of HIV-1 [21].

Taken together, there is strong evidence that exposure to heterologous cells and allogenic material alters the susceptibility of cells to lentiviral infection. However, whether this translates into reduced host infection or altered pathogenesis is less well understood. The role of cell exposure is particularly relevant when considering mucosal transmission. Not only is mucosa the major route of cell-free and cell-associated HIV-1 transmission [22-25], the vaginal and rectal mucosa is commonly exposed to heterologous cells and allogeneic material during sexual activity. Ejaculates contain HLA antigen expressing CD4+ T cells, macrophages, neutrophils, germ cells, epithelial cells and to some extent spermatozoa [26].

Given the reports of seronegativity in cohorts of sex workers with high-risk exposure [11], we hypothesized that HIV-1 transmission or progression could be altered by prior or concurrent immune stimulation by mucosal exposure to heterologous cells. We addressed this question directly using the FIV animal model of vaginal HIV-1 transmission. We repeatedly exposed cats by mucosal exposure to heterologous cells or media, assayed for lymphocyte phenotype as well as proliferative responses against cellular material, and then vaginally challenged cats with either cell-associated or cell-free FIV. We found that prior exposure to heterologous cells induced an immune response that was associated with reduced viral burden after mucosal challenge with cell-associated, but not cell-free, FIV.

## Methods

### Experimental design

To maximize genetic diversity, 22 female (Liberty Laboratory) and 6 male (Harlan Laboratories) SPF cats were obtained, housed, acclimated, and cared for in accordance with the standards of the American Association of Accreditation of Laboratory Animal Care and The Ohio State University Institutional Animal Care and Use Committee. Peripheral blood mononuclear cells (PBMC) from each female cat were tested against irradiated cells from each male cat in a one-way mixed lymphocyte reaction (MLR). The four male cats whose cells induced the highest MLR proliferation were used as sources of heterologous cells for this study. Female cats (n = 11 per group) were vaginally exposed once weekly for 12 weeks to lymphocyte media (Media) or 5000 male lymphocytes (Cells) in 50 μl final volume of lymphocyte media. For exposure inocula, male lymphocyte samples were not pooled. Female cats were exposed to heterologous cells from a different male cat each week for four weeks and then the cycle was repeated. One week after the 12^th ^exposure, three animals from each exposure group were euthanized to collect blood and tissue samples to evaluate tissue immune responses. Tissue samples were collected from the popliteal lymph node (PLN), mesenteric lymph node (MLN), medial iliac lymph node (ILN), small intestinal intraepithelial lymphocytes (IEL), and small intestinal lamina propria lymphocytes (LPL). One week after the 12^th ^exposure, the remainder of the cats from each group (n = 8) were vaginally challenged with cell-associated (n = 4) or cell-free (n = 4) FIV NCSU_1_. At 12 weeks post challenge (PC), blood, lymph nodes (popliteal, mesenteric, medial iliac) and gut (both IEL and LPL) were obtained at necropsy to quantify tissue viral load and examine immune responses. Mucosal exposure, viral inoculation, and venipuncture were performed under anesthesia induced by intravenous tiletamine and zolazepam (Telazol, Fort Dodge Animal Health, Fort Dodge, IA).

### Virus inocula

Cats were inoculated with either 5 × 10^5 ^FIV-infected Mya-1 cells (cell-associated challenge) or 50 × TCID_50 _tissue culture supernatant (cell-free challenge). Both inocula were infected with FIV-NCSU_1 _an A-clade FIV. To obtain cell-associated inocula, Mya-1 T-cells were cultured for three days in complete RPMI 1640 lymphocyte media containing 20% fetal bovine serum (Atlanta Biologicals, Norcross, Ga.), 1 mM sodium pyruvate, 0.1 mM Hepes buffer sodium, 5 × 10^-5 ^M β-2-mercaptoethanol, 100 U of penicillin/ml, 100 μg of streptomycin/ml (all from Invitrogen Life Sciences) and 100 U/ml recombinant human interleukin-2 (IL-2) kindly provided by the NIH AIDS Research and Reagent Program at 37°C 5% CO_2_, and then infected with 20 × TCID_50 _cell-free FIV-NCSU_1 _tissue culture supernatant. At day 5 post-infection, infected cells were harvested and frozen in liquid nitrogen until use, with aliquots saved to analyze the degree of infectivity by real-time PCR, co-culture with feline CD4+ indicator cells [27], immunocytochemistry and western blot. For immunocytochemistry, cells were incubated with 1/1000 of serum from a chronically FIV-infected cat at 37°C for 30 min, probed with goat anti-cat IgG-FITC (USB Corporation, Cleveland, OH), and fixed with 2% paraformaldehyde. Fluorescence was monitored by confocal imaging system LEICA TCS SP2 AOBS (Leica Microsystems, Exton, PA) and 98% of the cells were infected. This correlated with real-time PCR and co-culture assays indicating at least one in ten cells was infected. Cell lysates were probed with 1:1000 anti-gp120 (SU1-30) (Custom Monoclonals International, Sacramento, CA) to confirm expression of FIV Env [28].

### Sample processing

PBMC and lymphocytes from the distal jejunum, popliteal, mesenteric, and medial iliac lymph nodes were processed as previously described [29-31]. Intraepithelial lymphocytes (IEL) and lamina propria lymphocytes (LPL) were isolated from 10 inches of distal jejunum following excision of Peyer's patches and lymphoid follicles [30,31]. Cells were either used immediately (T-cell phenotype and proliferation assays) or were washed, treated, and pelleted (DNA, RNA or protein extraction).

### Real time (RT)-PCR

DNA was purified from PBMC and tissue lymphocytes using DNeasy Tissue Kit (Qiagen, Valencia, CA). A conserved region (170-bp) of FIV-gag was amplified from 150 ng of each sample with primers GagNCSU_1_-1247 sense (5'-GCTTAAAGCAATTGACGGCAGAGTATGATCG-3') and GagNCSU_1_-1417 anti-sense (5'-CCTCGAGATACCATGCTCTACACTGCATCC-3') as previously described [28]. Proviral copies were expressed as gag copies per million GAPDH copies [31]. The sensitivity of this assay is 10 copies FIV per μg of DNA [32]. Reaction mixtures containing 2 × SYBR-Green master mix (Qiagen, Valencia, CA), 0.5 μM primers, and 2-5 μl of each DNA sample were amplified in a 96-well plate (ABgene, Rochester, NY) using the Mx3000 (Stratagene, La Jolla, CA) and the following PCR conditions: 15 min at 94°C then 40 cycles of 30 seconds at 94°C; 1 minute at 60°C, and 30 seconds at 72°C, followed by one cycle of 1 minute at 95°C and 30 seconds at 55°C. Standard curves were generated for primer pairs using serial dilutions of the following plasmids: pCR2.1-GAPDH and pCR1-gag [31].

### Reverse transcriptase RT-PCR

RNA was extracted from 200 μl of plasma using High Pure Viral RNA Kit (Roche, Indianapolis, IN). Reverse transcription RT-PCR was performed in triplicate wells of a 96-well plate (ABgene, Rochester, NY) as one step RT-PCR in 25 μl containing 2-3 μl of purified RNA, 0.5 μM QuantiTect RT Mix, 2 × SYBR-Green master mix (Qiagen, Valencia, CA), and 0.5 μM primers. Following 30 minutes at 50°C for reverse transcription, program conditions were as described above for RT-PCR. The detection limit of this assay is ≤ 10 copies per 50 μl of plasma and is similar to the findings of others [33].

### Flow cytometric analysis

Absolute lymphocyte counts were calculated using an automated white blood cell count (VetSCAN HMT, Abaxis, Union City, CA) and manual differential. T-cell subsets were analyzed by FACS Calibur (Becton Dickinson, San Jose, CA) as previously described [29,30].

### Lymphoproliferation

Lymphoproliferation was assayed in PBMC prior to exposure and in PBMC and tissue lymphocytes at 12 week post exposure (PE). Cells (1 × 10^5^) were incubated in triplicate in one of four conditions: lymphocyte media alone, 5 μg/ml of concanavalin A (Con A), irradiated cells as a mixed lymphocyte reaction (MLR), or whole cell lysate (WCL) at 37°C, 5% CO_2 _for 4 days [34]. Irradiated cells and whole cell lysate were obtained from male donor (male), the cat's own PBMC (self), or the cells used for cell-associated FIV challenge (Mya-1). Irradiated cells for the MLR were obtained by irradiating cells in a T-25 flask or 6 well plates at a final concentration of 1 × 10^6^/mL in lymphocyte media for 85 minutes @ 7500 rad in a Gammacell 40, cs-137 irradiator. Whole cell lysate (WCL) was prepared by sonicating the cells for 1 minute/5 mL @ 22 μM amplitude. Protein content was quantified using the Bradford reagent. Wells were pulsed with 1 *μ*Ci/well of tritiated thymidine (MP Biomedicals Corp., Irvine, CA) for 18 hrs and then harvested using a FilterMate Cell Harvester (PerkinElmer Life Sciences, Downers Grove, IL). Uptake was quantified as counts per minute (CPM) using a MicroBeta Jet Liquid Scintillation and Luminescence counter (Wallac, Turku, Finland). Triplicates were averaged and the proliferation index (PI) was calculated: (Ave CPM at particular time point/Ave background CPM at same time point)/(Ave. CPM at pre/Ave background CPM at pre) × 100. Pre exposure data was normalized to 100% for each animal and data presented as change from baseline.

### ELISA

Serum antibody against FIV p24Gag was detected by adding serial dilutions of serum to microtiter plates (Immulon 2 HB, Dynex Technologies Inc., Chantilly, VA) coated with 0.1 μg/well p24Gag fusion protein as previously described [35]. Titers were expressed as the inverse of the highest dilution that produced an OD ≥ 0.1 and ≥ 3-fold the OD of the animal's pre-study sample.

### Statistical analyses

Flow phenotyping results were analyzed using analysis of covariance for PBMC and multivariate analysis of variance for tissue samples with separate models for each T-cell subset. For PBMC, differences between the Heterologous and Media groups were tested with the baseline percentage of the T-cell subset included as a covariate. For tissue sample results, the endpoints were the percentages of the T-cell subset in each of the five tissue sources (PLN, MLN, ILN, IEL, and LPL). Separate residual variances were estimated for each tissue source. Contrasts within the model evaluated differences separately between Heterologous and Media groups for each type of tissue.

For MLR and WCL analyses, the endpoint was a cell PI measuring change from baseline. Linear mixed models were used to analyze differences in mean cell PI between the treatment groups and between antigen types (self, male, Mya-1). Also, for the post-challenge model, the challenge types (cell-free, cell-associated) were compared. PBMC models included a covariate for the baseline CPM ratio and a random subject effect. Tissue sample models were multivariate and estimated separate residual variances and random subject effects for each tissue type. Log-transformed PI values were used in the analyses in order to correct for skewness. All statistical tests were evaluated at the two-sided alpha = .05 significance level. Analyses were performed using SAS^® ^version 9.2 (The SAS Institute, Cary, NC).

Viral loads were used to calculate the arithmetic mean for different treatment groups and then compared using a Student's t-test with differences at p < 0.05 regarded as significant.

## Results

### Mucosal exposure to heterologous cells induces systemic and mucosal lymphocyte phenotype shifts

**Figure 1:**
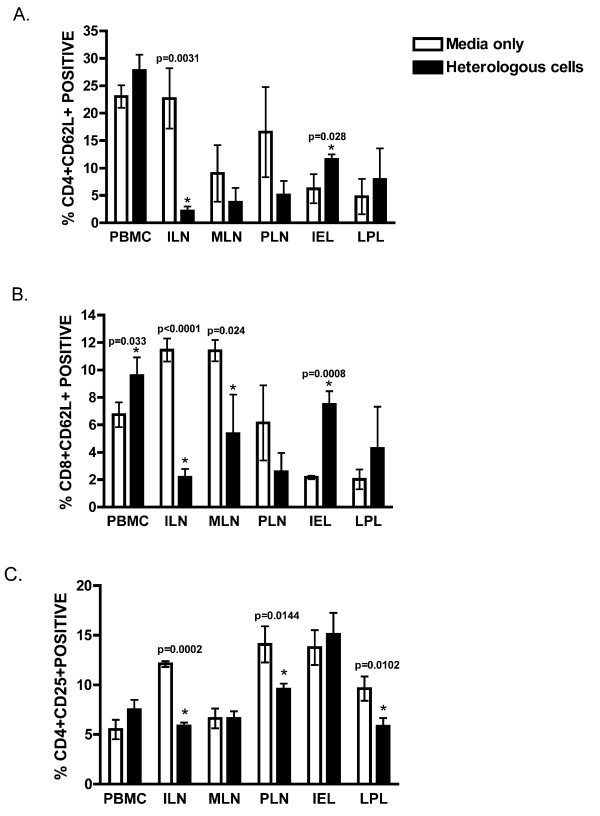
**Percentage of (a) CD4+, and (b) CD8+ cells expressing L-selectin; and (c) CD4+25+ cells**. Data present comparison of these cells in various tissues between media and heterologous cell exposed subjects Data presented as arithmetic mean, SD. * Statistically significant (p < 0.05) between Media and Heterologous groups for that tissue.

To determine whether repeated mucosal exposure to heterologous cells resulted in shifts of T cell subsets and activation status, blood and lymphoid tissues were examined by flow cytometry. No changes were detected in the total percentage of CD4+ or CD8+ T cells in the blood and lymphoid tissue (data not shown). L-selectin (CD62L) was examined as a marker primarily found on naïve lymphocytes as well as some central memory T cells [36-39]. Loss of L-selectin expression has previously been shown to correlate with FIV antiviral activity [40]. Following repeated mucosal exposure to heterologous cells, the percentage of CD4+ T cells expressing L-selectin were increased in the gut tissue, IEL (p = 0.028) and decreased in iliac lymph node (p = 0.0031) (Fig 1a). CD8+ T cells expressing L-selectin were increased in the blood (p = 0.033) and the gut tissue (IEL, p = 0.0008) but were decreased in the lymph nodes (ILN, p < 0.0001; MLN, p = 0.024) (Fig 1b)

As is seen in other species, feline Tregs are predominantly CD4+CD25+ cells; and FoxP3 expression has recently been shown to correlate with dual CD4 and CD25 expression by lymphocytes [41]. Following repeated mucosal exposure to heterologous cells, the percentage of CD4+CD25+ T-cells was significantly decreased in the iliac LN (p = 0.0002), popliteal LN (p = 0.0144), and intestinal LPL (p = 0.0102) while no changes were noted in blood, mesenteric LN, or intestinal IEL (Fig 1c).

### Mucosal exposure to heterologous cells induces lymphoproliferative responses against heterologous cells

**Figure 2:**
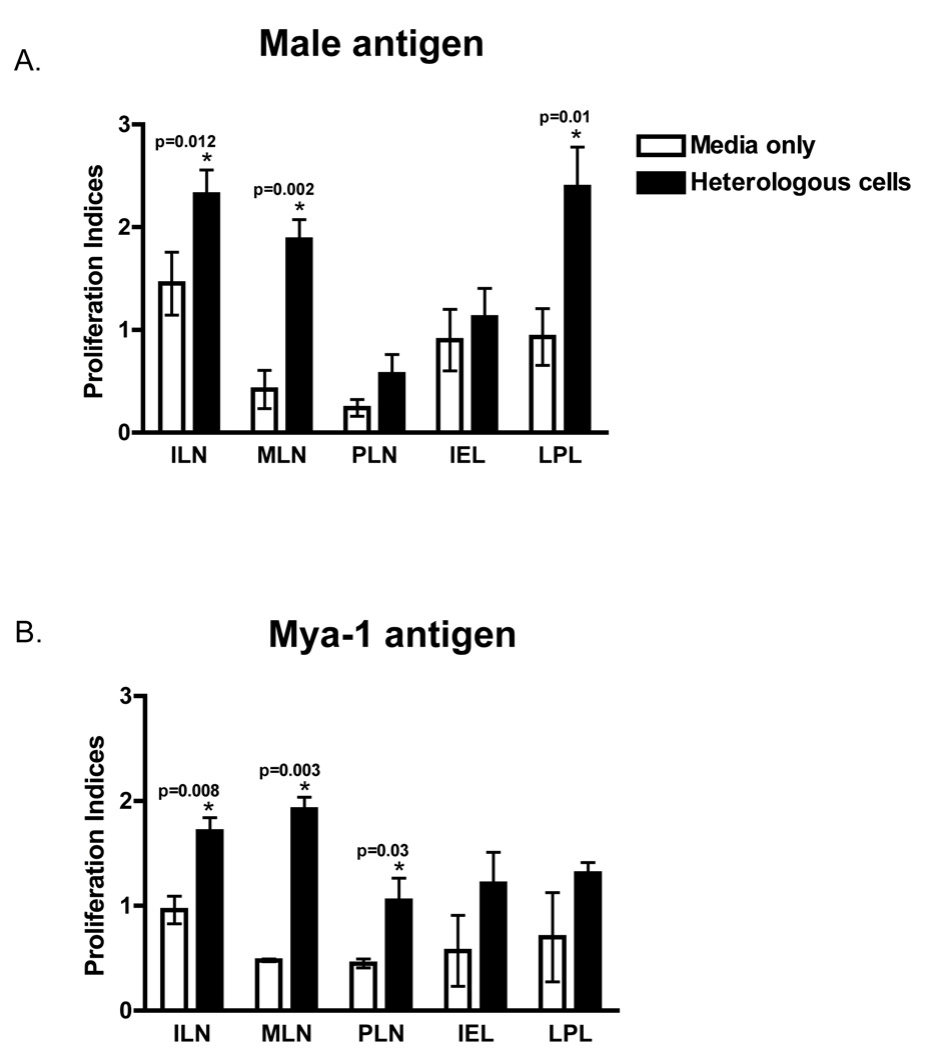
**Mixed lymphocyte response against (a) male and (b) Mya-1 T cells in lymph nodes and gut**. The data represent response after 12-weeks of repeated mucosal exposure to heterologous cells or media alone. Data are presented as arithmetic mean, SD cell proliferation index measuring change from baseline. * Statistically significant (p < 0.05) between Media and Heterologous groups for that tissue.

Exposure to heterologous cells for 12 weeks induced a MLR response against male and/or Mya-1 irradiated cells in multiple mucosal and systemic sites. Significant responses against male cells were detected in lymphocytes from the ILN, MLN, and LPL (ILN, p = 0.012; MLN, p = 0.002; LPL, p = 0.01) while significant responses against Mya-1 cells were only detected in the lymph nodes (ILN, p = 0.008; MLN, p = 0.003; PLN, p = 0.03) (Fig 2). Consistent with the naïve (CD62L+) phenotype found in blood, a significant proliferative response was not detected in PBMC (data not shown). Proliferation against whole cell lysate (WCL) of male cells only was detected in the ILN (p = 0.004, data not shown). Significant proliferation to WCL was not detected elsewhere.

### Provirus burden is reduced following cell-associated mucosal challenge after previous exposure to heterologous cells

After repeated exposure to either media or heterologous cells, cats were vaginally challenged with cell-associated or cell-free FIV and viral load was measured after 12 weeks. FIV-RNA was consistently detected in the plasma of all cats challenged with FIV (Table 1 &2) and was similar in animals challenged with cell-associated (Table 1) or cell-free FIV (Table 2). Provirus was detected in all animals but in cats challenged with cell-free FIV, copy numbers were lower, were not detected in all tissues, and there were no significant differences detected between the Media and Heterologous treatments (Table 2).

**Table 1:** Cell-associated FIV challenge summary

Cat ID	gag copy/million GAPDH						gag copy/mL	Anti-FIV p24 IgG dilution
**Media only**	**PBMC**	**ILN**	**MLN**	**PLN**	**IEL**	**LPL**	**PLASMA**	**SERUM**
AMX1	15585	9135	4899	7366	1072	68	73	12800
AMY5	83572	37693	29756	65000	3062	538	69	102400
ANG5	29819	21413	10079	34634	3700	173	63	6400
ANE5	2313	6737	2683	6100	1815	NA	61	400
**MEAN**	**32822**	**18745**	**11854**	**28275**	**2412**	**260**	**67**	**30500**
								
**Heterologous cells**								
ANZ4	1265	2261	116	887	0	5	44	6400
QNY5	2251	9563	2255	9225	1603	NA	83	800
AMY6	20177	22633	7848	20530	3796	5	70	12800
AMW5	5477	11740	3434	9716	0	44	26	6400
**MEAN**	**7293**	**11549**	**3413**	**10089**	**1350**	**18**	**56**	**6600**

**Table 2:** Cell-free FIV challenge summary

Cat ID	gag copy/million GAPDH						gag copy/mL	Anti-FIV p24 IgG dilution
**Media only**	**PBMC**	**ILN**	**MLN**	**PLN**	**IEL**	**LPL**	**PLASMA**	**SERUM**
IMO4	1299	1495	0	762	0	0	51	200
QNV2	1674	7905	0	1544	0	0	50	0
ANC6	1579	3676	0	1153	0	0	62	0
AMW6	2035	2554	0	973	0	0	48	200
**MEAN**	**1647**	**3907**	**0**	**1108**	**0**	**0**	**53**	**100**
								
**Heterologous cells**								
AMY3	1877	2927	543	2595	285	3	50	6400
AMW3	1372	4514	0	1694	0	0	56	0
AMX3	1554	3767	0	NA	NA	0	51	100
QNU6	1606	2551	0	NA	886	0	25	0
**MEAN**	**1602**	**3440**	**136**	**2144**	**390**	**1**	**45**	**1625**

**Figure 3:**
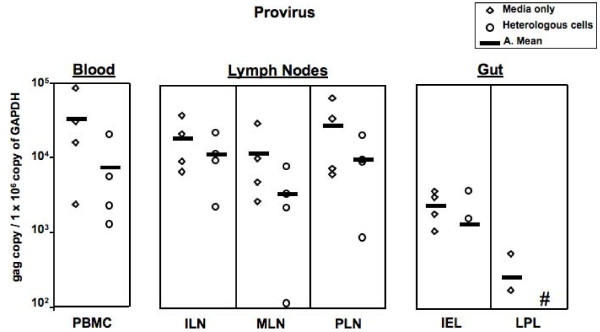
**Proviral burden 12 weeks post-challenge with cell-associated FIV in cats previously exposed to heterologous cells/media**. Proviral burden was approximately 1 log lower in the Heterologous group. # Less than 100 copies detected. In the LPL, one sample in each group was damaged during processing and therefore unavailable. Within the heterologous group, both the IEL and LPL had two different animals with the same copy number; for IEL 0 copies were seen in two animals, for LPL 5 copies.

In contrast, in cats challenged with cell-associated virus, PBMC and tissue proviral burdens were approximately a log lower in animals previously exposed to heterologous cells (Fig 3) as compared to media controls. The same trend of reduced viral load was detected in blood samples taken 8 weeks post challenge (data not shown).

There was no detectable response to the p24Gag fusion protein after exposure to heterologous cells (or media) and prior to challenge. Antibody response was only detected after viral challenge. IgG against p24 was readily detected in all cats infected with cell-associated FIV (Table 1) but was undetectable (n = 4) or minimal (titer of 100-200 n = 3) in the majority of cell-free challenged cats (Table 2).

## Discussion

This study was designed to mimic what might occur in women exposed to multiple sexual partners and determine whether prior mucosal exposure to heterologous cells could alter lentiviral transmission or disease. In the study described here, cats repeatedly exposed to heterologous cells had reduced proviral burden when compared to cats receiving media alone following mucosal challenge with cell-associated but not cell-free FIV. While the final numbers of animals in each challenge group were relatively small (n = 4), we and others have demonstrated relevant challenge findings in the FIV and SIV animal models with final group sizes in this range [42-44]. In addition, cats mucosally exposed to heterologous cells had shifts in lymphocyte activation as well as proliferation against cellular antigens, including cells (Mya-1) that they had not been previously exposed to. This suggests that FIV infection was modulated by responses against the infected cells, not simply FIV, as no differences were seen between the groups following challenge with cell-free FIV.

Mucosal exposure to heterologous cells resulted in a reduced percentage of L-selectin positive in the LNs with a concurrent expansion of L-selectin positive cells in the blood and gut. L-selectin (CD62L) is expressed on naive CD4+ T cells as well as a small subset of memory T cells and facilitates immune surveillance by enabling the cells to recirculate and compartmentalize between blood and lymph node [36-39]. FIV and HIV infections are associated with progressive immune dysfunction with reduced capacity to respond due to poor response to recall antigens [45-48]. L-selectin expression may thus affect HIV-1 pathogenesis by altering the ability to recall antigens. It may also play a role in HIV-1 pathogenesis by enhancing virus transmission to CD4+ T lymphocytes [49] possibly due to enhanced expression of surface CXCR4 in lymphocytes through L-selectin signaling have been suggested [50]. Hence, changes in L-selectin expressed cells after mucosal exposure may be responsible for the observed alteration of susceptibility to FIV infection.

Changes in L-selectin expression after mucosal exposure were most evident in the iliac LN (CD4+62L+, p = 0.0031; CD8+62L+, p < 0.0001) and IEL populations (CD4+62L+, p = 0.028; CD8+62L+, p = 0.0008). The marked ILN changes are likely attributable to the site of exposure as the ILN drains the cervicovaginal mucosa. Previously, we have shown that the ILN and IEL lymphocyte populations are uniquely altered in chronic FIV infection [51] indicating that the immune function and interactions at these sites should be further investigated in multiple model systems.

In the LN, the shift to an activated phenotype occurred concurrently with functional lymphocyte activation as measured by proliferation whereas the LPL response was enhanced without a detectable expansion of activated cells. This may be related to the high percentage of activated cells normally present in the gut [52]. Interestingly, the MLR response that developed following exposure to heterologous cells was also cross-reactive against cells that the animals had not been exposed to, namely the Mya-1 cell line. This cross reactive immune response was likely responsible for the decrease in viral burden seen following subsequent challenge with FIV infected Mya-1 cells but not CF FIV.

The shift towards an activated phenotype and concurrent proliferative response was also associated with a reduction in the percentage of CD4+CD25+ cells in the ILN (p = 0.0002), PLN (p = 0.0144), and LPL (p = 0.0102). Feline CD4+CD25+ cells have been previously shown to have regulatory activity ex vivo and in vitro [53]. FoxP3, a marker of Treg activity, increases in CD4+CD25+ T cells following FIV infection [41,54]. While we did not measure regulatory function and FoxP3 reagents were not available at the time of this study, the data suggests that a reduction of the number of T-regulatory cells within the environment was associated with the development of an effector immune response. Tregs may play a crucial role in immunopathogenesis of many viral infections including herpes simplex virus (HSV), hepatitis C virus (HCV), Friend virus and lentiviruses including FIV [53-58]. These studies suggest that activation of Tregs following viral infection leads to suppression of CD4+ and CD8+ effector responses resulting in decreased viral clearance and consequently establishment of infection. In-vitro studies suggest that Treg cells from HIV-infected subjects suppress the effector function of CD8+ antigen-specific HIV gag responses [59,60]. Though mechanisms involved in suppression of infection by Treg depletion remain unclear, few studies have suggested granzyme, CTLA-4, and tumor growth factor (TGF-beta), chemokine and cytokine regulation [61-63]. Previous allograft studies suggest the role of Treg cells in inhibiting alloimmune responses [64,65].

In contrast to the strong MLR response detected against whole cells at multiple sites, we did not find significant proliferation against cell lysate after heterologous cell exposure except in the ILN. This response was likely confined to the draining lymph node due to rapid degradation however a unique response against intracellular antigens cannot be excluded. Taken together, these data suggest that the immune activation and relative protection induced by exposure to heterologous cells was due to responses against cell surface antigens versus intracellular antigens and is consistent with findings that intact stimulating cells are required for efficacious production of anti-HIV activity [66]. Whether this is due to conformational interactions, interaction with secondary molecules, or a combination has relevance for vaccine and small molecule therapy and remains an area for investigation.

Although neutralizing antibodies were not measured in this study, previous reports support the potential for a protective role by the humoral arm of the immune system. Vaccination of macaques with uninfected human cells were found to be protected against SIV grown in the same cell type and protection was correlated with the detection of anti-HLA class I antibodies [5]. Because viral gp120 and HLA share a degree of homology [67] it has been hypothesized that anti-HLA antibodies may neutralize free virions by binding to HLA or gp120 on the viral envelope leading to cross-reactive protection. Given recent detection of antibodies against cell line antigens in cats vaccinated with commercially available vaccines generated in this cell line, the contribution of cross-reactive humoral responses is clearly worth further investigation [68].

In this study, cats previously exposed to heterologous cells and then challenged with cell-associated FIV had lower tissue proviral burdens than those had been previously only exposed to media. This was not true for cell-free FIV, a finding also supported by a recent study [69]. Taken together; these studies strongly suggest that intact cellular proteins are critical for induction of protective immune responses against infected cells. As cell-associated virus appears to have an important role in HIV sexual transmission [70-73], our findings are encouraging as exposure to heterologous male lymphocytes induced cross-reactive proliferation against the challenge inoculum cells suggesting that cross reactivity plays a significant role in allo-induced antiviral activity. An additional control could have been to examine the effect of mucosal exposure to the challenge cells (Mya-1). It is reasonable to speculate that prior exposure to uninfected Mya-1 cells would induce the strongest protection to FIV-infected Mya-1 cells, however it is also possible that exposure to a uniform cell line may have induced immune responses that varied markedly from what would be seen following exposure to a heterogeneous 'normal' lymphocyte population as we examined in this study.

Under natural conditions, biting appears to be the most efficient mode of FIV transmission. Virus is easily isolated from the saliva of infected cats and both experimental and epidemiological studies suggest that a single bite exposure is sufficient to transmit the virus from an infected to a naive cat [74]. Infectious FIV is present in genital secretions from naturally and experimentally infected male cats [75-77]. Replication-competent FIV is found in both the cell-free fraction and cell- associated fraction of semen and can be transmitted to cats by a single uterine artificial insemination [75,77].

As an animal model, mucosal transmission of FIV has been developed extensively. FIV transmission mimics the diversity apparent in HIV-1 vaginal transmission and virus strains representing at least three subtypes of FIV can be transmitted across the vaginal, rectal, or oral mucosa by cell-associated as well as cell-free virus [34,78-83] similar to reported previously [84,85,72], mucosal exposure to cell-associated FIV resulted in greater virus burden than did exposure to cell-free FIV, particularly in the gut. This could be due to dose effect. However, there is no gold standard to directly correlate in vitro cell-free and cell-associated titrations with each other or to predict in vivo outcomes. In addition, a recent study suggests that mucosal dose alone is not predictive of infection outcome [86].

While exposure to heterologous cells did not provide complete protection, the log reduction in proviral burden after cell-associated challenge is similar to the reduction in virus burden reported in vaccine studies [2-8]. Because early viral set point and higher virus burden predict a more rapid progression of HIV infection [87,88], containing the virus early on appears to alter the downstream progression to disease that can have significant effects on patient health as well as potential transmission to others. The results reported here are particularly significant given the high-dose challenge used in this study. Although high-dose challenges are commonly used in animal model studies, lower doses more likely mimic what is seen in HIV-1 exposure, particularly sexual exposure. As mucosal administration of low-dose cell-associated FIV results in non-progressive infection (Burkhard unpublished data) or viral latency [28], further reduction of infection by a log under low-dose exposure circumstances may increase the threshold for infection resulting in local or 'silent' infection as has been reported in certain high-risk cohorts [89-92].

## Conclusion

Multiple mechanisms have been proposed for the lack of infection in HIV-1 exposed but seronegative individuals including viral determinants of infection, low-level HIV-1 cellular and humoral immune responses, and genetic variation. However, the phenomenon remains poorly understood. Several SIV and FIV vaccine studies, as well as human epidemiologic data, suggest that alloimmune responses may be associated with protection against lentiviral infection [2-8]. We examined the role of vaginal exposure to heterologous cells on lentiviral infection by using the FIV model of mucosal transmission. We found that repeated exposure of the mucosa to heterologous cells led to functional and phenotypic activation of immune system. This suggests that the threshold for mucosal infection by infected cells or the early pathogenesis can be modulated by immune responses against heterologous cells. This is further supported by the absence of differences in viral burden between those challenged with CF virus. Overall, our data suggests that mucosal exposure to non-viral antigens may induce cross-reactive immune responses that can reduce virus burden. While these may not be completely protective, the ability to reduce early viral set points has significant implications for further transmission and disease progression and hence may provide additional strategies for therapy and vaccination. Our findings support a need to reexamine the role that immune responses against heterologous and allogeneic cells may play in HIV-1 transmission, early infection, viral dissemination, and progression to disease.

## Competing interests

The authors declare that they have no competing interests.

## Authors' contributions

SBK, SEL and MJB were responsible for the design of the study. SBK and MJB were responsible for the draft of the manuscript. SBK performed most of experiments. SEL performed flow cytometry experiments. BA helped in isolation of IEL, IPL and provided FIV viral stocks. KP performed statistical analysis. All authors read and approved the final manuscript.
